# Searching the Internet for Infertility Information: A Survey of Patient Needs and Preferences

**DOI:** 10.2196/15132

**Published:** 2019-12-12

**Authors:** Felicia Brochu, Stephanie Robins, Skye A Miner, Paul H Grunberg, Peter Chan, Kirk Lo, Hananel E G Holzer, Neal Mahutte, Sophia Ouhilal, Togas Tulandi, Phyllis Zelkowitz

**Affiliations:** 1 Department of Psychiatry McGill University Montreal, QC Canada; 2 Department of Psychiatry Jewish General Hospital Montreal, QC Canada; 3 Lady Davis Institute Jewish General Hospital Montreal, QC Canada; 4 Department of Sociology McGill University Montreal, QC Canada; 5 Department of Psychology McGill University Montreal, QC Canada; 6 McGill University Health Center Montreal, QC Canada; 7 Mount Sinai Hospital Toronto, ON Canada; 8 Hadassah University Hospitals Jerusalem Israel; 9 Hebrew University Jerusalem Israel; 10 Montreal Fertility Centre Montreal, QC Canada

**Keywords:** infertility, internet, consumer health information, patient satisfaction, stress, psychological

## Abstract

**Background:**

Given the complexity of infertility diagnoses and treatments and the convenience of the internet for finding health-related information, people undergoing infertility treatments often use Web-based resources to obtain infertility information and support. However, little is known about the types of information and support resources infertility patients search for on the internet and whether these resources meet their needs.

**Objective:**

The aims of this study were to (1) examine what individual factors, namely, demographic characteristics and distress, are associated with searching the internet for different types of infertility-related information and support resources and (2) determine whether Web-based resources meet the needs of patients.

**Methods:**

Men and women seeking infertility care responded to a survey assessing use of Web-based resources for accessing infertility-related information and support. The survey further assessed satisfaction with Web-based resources as well as perceived stress and depressive symptomatology.

**Results:**

A total of 567 participants, including 254 men and 313 women, completed the survey. Most participants (490/558, 87.8%) had searched the internet for infertility information and support. Searchers were more likely to be women (*P*<.001), highly educated (*P*=.04), long-term patients (*P*=.03), and more distressed (*P*=.04). Causes of infertility, treatment options, and scientific literature about infertility were the three most frequently searched topics, whereas ways to discuss treatment with family and friends as well as surrogacy and ways to find peer support were the three least searched topics. Of those who searched the internet, 70.9% (346/488) indicated that their needs were met by Web-based information, whereas 29.1% (142/488) said that their needs were not met. Having unmet needs was related to greater levels of perceived stress (*P*=.005) and depressive symptomatology (*P*=.03).

**Conclusions:**

This study provides evidence for the important role of the internet in accessing infertility information and support and for the ability of Web-based resources to meet patients’ needs. However, although distressed patients reported particularly high rates of searching, their needs were not always met, suggesting that they may benefit from alternative sources of information and support or guidance from health care providers when searching the internet.

## Introduction

### Background

The widespread access to internet technologies (eg, computers and mobile phones) has facilitated the delivery and acquisition of health information and support. Approximately 70% of North American adults use the internet as a source of health information [1,2], with higher rates among people with health concerns as well as those who rate their health as poor [3,4]. Web-based resources offer a number of advantages over print and face-to-face resources; these include ready availability, instantaneous access to frequently updated sources of information, anonymity, and contact with people with similar difficulties. Furthermore, the internet may offer an opportunity to increase access to health resources by overcoming geographic and socioeconomic barriers that can sometimes prevent underserved populations, such as infertile men and linguistic minorities, from obtaining information and support. Accordingly, people with infertility concerns may seek out information and emotional support using the internet.

Infertility, defined as the inability to achieve clinical pregnancy following 12 months of regular unprotected intercourse [5], has an estimated Canadian prevalence between 11.5% and 16% [6]. Infertility is a chronic health condition that is associated with feelings of stigma, loss of control, and *a rollercoaster of emotions* [7,8]. To gain familiarity with the medical language used in the diagnosis and treatment of infertility and to better cope with the potential stigma and psychological distress, people with infertility concerns are likely to search the internet to find infertility-related information and social support [9-11]. Accordingly, a systematic review revealed that patients with infertility tend to go on the internet to meet their informational needs, find emotional and psychological support, and find assistance for medical decision making [12]. After accessing Web-based sources of information, infertile women have been shown to feel better informed and better able to make infertility-related decisions [10]. Previous research has also demonstrated how the internet may be a useful tool to provide educational and emotional support to those living with a stigmatized health condition [13]. The use of online support groups has been associated with a number of advantages including increased patient empowerment [14,15], normalization of patients’ experiences [16,17], and reduced social isolation [18]. These benefits suggest that online discussion about emotions and personal experiences [17,19] may be helpful for improving patients’ psychosocial experience of infertility.

Notwithstanding the potential benefits of online communication, evidence suggests that the use of the internet poses risks of misinformation and increased emotional strains to infertility patients [12,20]. Examples of emotional consequences include feelings of sadness and distress after reading both negative and positive stories relating to others’ treatment outcomes, obsessive use of online communities, and misunderstandings among group members [21]. Furthermore, using the internet to access health information involves challenges such as difficulty locating or accessing the information, judging the quality and comprehensiveness of sources, and understanding technical terms [22,23]. Studies investigating the quality of Web-based infertility-related information suggest that available websites generally do not meet standards for readability, quality, and suitability [24,25]. Varying levels of electronic health (eHealth) literacy, which is the ability to search, find, understand, and appraise health information found on the internet [26], can exacerbate the difficulties to access resources for one’s needs.

However, few studies have investigated whether the information and support resources available on the internet in fact meet patients’ needs and expectations [12]. Moreover, one study found that only half of their sample of women with infertility issues who used an online expert forum to discuss infertility were satisfied with the responses received [27]. Although this study highlights the potential of Web-based resources to meet users’ needs, previous research is limited in studying online communities who may not represent the diverse infertility patient population. Research has also primarily focused on the experiences of infertile women, whereas less emphasis has been given to the needs and experiences of men. More research is needed to gauge the impact of Web-based resources other than forums on the needs of diverse infertility patients, especially men who tend to be underrepresented in infertility care and research [28,29] and whose needs for information and support may be different from those of women.

Individual characteristics such as gender may be related to the tendency to search for Web-based infertility-related information. Gender differences in help–seeking [30,31] and health information–seeking behaviors [32] extend to searches for health information on the internet. Specifically, past studies indicate that women engage in more Web-based health information seeking [33-35], indicating that infertile women may be particularly likely to search the internet for infertility information and support. Furthermore, in addition to enduring the physical burden of infertility, women more often report infertility-related emotional distress [36]. Perhaps, for this reason, infertile women generally seek more social support [37]. Research shows that women are generally more active in online communities compared with men [21,38], although there is some evidence that men also benefit from interacting online [39-41].

Other individual characteristics found to be associated with the use of Web-based health resources include white non-Hispanic ethnicity, higher educational attainment, higher income, and frequent internet use in general [42,43]. Language and health literacy difficulties can reduce accessibility to Web-based health information and support, thereby making it challenging for immigrants [44] and people with less education [45] to access the appropriate resources for their needs. In addition, greater psychological distress may encourage more Web-based health information seeking and the use of more online sources of support [46,47]. Similarly, individual characteristics are likely to influence whether Web-based health information meets people’s needs. Specifically, nonwhite ethnicity and lower income have been shown to be related to greater perceived helpfulness of infertility-related Web-based resources [10], emphasizing the need to explore differences in how people use and perceive Web-based health resources in a diverse group of individuals with infertility concerns.

Given the omnipresence of the internet in the lives of infertility patients, efforts should be made to better understand patients’ online experiences. This study addressed an important gap in the literature by investigating how both men and women seeking infertility care use the internet and whether they are satisfied with what they find. By understanding the online experiences of patients from diverse sociodemographic backgrounds, we may be better able to characterize the types of information and support resources that people need and analyze whether the internet is useful in meeting those needs. Such information is necessary to guide the development of future Web-based resources that are tailored to the needs of infertility patients.

### Study Objectives

The first objective of this study was to examine whether people seeking infertility care searched the internet for infertility-related information and support resources and to explore whether demographic and psychological factors were associated with searching. Specifically, gender, immigration status, education, treatment duration, perceived stress, and depressive symptomatology were examined in relation to searching the internet. The second objective was to describe the infertility-related topics that people endorsed searching for and to explore the relationships between types of searches and participant characteristics. Finally, this study aimed to inquire whether Web-based resources met people’s informational and support needs and to examine which participant characteristics were associated with satisfaction with Web-based resources.

## Methods

### Procedure and Participants

A self-report survey was distributed to men and women seeking infertility care services to investigate their needs and preferences regarding information and support about infertility issues. Recruitment was conducted from July to December 2015 in four fertility and urology clinics in the Montreal and Toronto areas. Participants were recruited in both private and hospital-based fertility clinics to ensure a sociodemographically diverse sample. Participants were eligible to participate if they were (1) seeking care at a fertility clinic, (2) at least 18 years old, (3) able to read and answer questions in French or English, and (4) able to access the internet. Of the 808 patients approached, almost all (795/807, 98.4%) were eligible. Only 6.2% (49/710) patients refused because of lack of interest, time constraints, and personal stress level or because they were unprepared to discuss infertility experiences. Those who agreed to participate in the study (n=746) accessed the Web-based survey on a tablet while sitting in clinicians’ waiting rooms, or through an emailed link that directed them to a secured website where they could complete the survey at a later time. Consent was implied if the participant accessed the link. Following completion of the study, participants received a Can $10 gift card. A total of 88.3% (659/746) of people who agreed to participate completed the survey. This study focused specifically on the experiences of men and women undergoing infertility treatment as a dyadic unit and thus reported data from participants in heterosexual relationships only (n=567). Respondents who were single (n=23), nonheterosexual (n=28), or who did not indicate their relationship type (n=89) were excluded from this analysis because their small number did not allow to appropriately investigate their distinct needs and experiences.

The study protocol was approved by the research ethics boards of all the institutions where recruitment took place.

### Materials

#### Patient Survey

The patient survey (Multimedia Appendix 1) was developed by the corresponding author in collaboration with coinvestigators and community stakeholders including reproductive health specialists and infertility patients. The survey was pilot tested to ensure the clarity and relevance of the items, the appropriate order of the questions, and the length of time required to complete the questionnaire. Feedback from stakeholders guided the final version of the questionnaire. The survey was sent via email; data were then anonymized by removing all personal information in the final dataset. The survey consisted of questions addressing patients’ experiences and opinions toward different sources of information and support as well as questions regarding fertility history and psychological and sociodemographic characteristics (eg, income and ethnicity). The survey assessed the use of Web-based resources for accessing infertility-related information and support. This section of the survey included three questions: (1) *Have you searched online for information about infertility?* to which participants answered yes or no; (2) *Did you look online for the following...?* (eg, scientific literature about infertility, diagnostic tests, and how to find peer support or mentor), where respondents selected all types of information and support resources that applied to them; and (3) *In general, did the information you found online meet your needs?* to which participants answered yes or no.

For the purpose of analysis, individual items listed in question 2 were grouped into four conceptual categories: *diagnostic information* (diagnostic tests, interpreting results of diagnostic tests, my diagnosis, scientific literature about infertility, and causes of infertility), *treatment information* (treatment options, success rates of treatment, medications used in treatment, side effects of treatment, adoption/foster parenting, using donor sperm or eggs, and surrogacy), information about *services and providers* (clinics where treatment is offered, my physician or medical team, coverage by provincial health care plans, coverage by private health care plans, and how to get a second opinion), and *connections with others* (others’ experiences with infertility, how to discuss treatment with family/friends, and how to find peer support/mentor). The categories were created by summing the number of specific items that heuristically fell in that category.

#### Depressive Symptomatology

The 2-item Patient Health Questionnaire [48] was used to assess the level of depressive symptoms patients had experienced in the previous 2 weeks. Each item is rated on a 4-point Likert-type scale ranging from 0 (not at all) to 3 (nearly every day). The score for each item is summed to obtain a total score between 0 and 6, with higher scores indicating more severe depressive symptomatology. A score of ≥2 warrants further clinical investigation for depression. The scale was shown to be comparable with its longer version (9-item Patient Health Questionnaire) and other depression scales in terms of reliability (Cronbach alpha=.83) and criterion and construct validity [48,49].

#### Perceived Stress

Perceived stress was evaluated using the 4-item Perceived Stress Scale [50]. Each item asks participants to rate the extent to which they experience life events as stressful and whether they feel able to overcome difficult events on a Likert-type scale ranging from 0 (never) to 4 (very often). Total scores vary between 0 and 16, with higher scores corresponding to greater perceived stress. The 4-item Perceived Stress Scale is normally distributed [51] and has demonstrated satisfactory reliability (Cronbach alpha=.77) and strong criterion validity [50,51].

### Data Analysis

Descriptive statistics were used to describe sample characteristics, including sex, age, annual household income, education, immigrant status, treatment duration, and causes of infertility. Descriptive statistics were also used to determine how many participants searched the internet for information and support resources and how many reported that this information met their needs. Bivariate analyses including Chi-squares and *t* test statistics were then performed to examine differences between those who did and did not search the internet (ie, searchers and nonsearchers) and those who reported that their information needs were or were not met. Furthermore, correlations, *t* tests, and analysis of variance were used to explore the relationship between individual characteristics and searching for each of the four information categories. Post hoc Hochberg GT2 tests were used to further investigate difference between groups. Variables that were significantly related to searching the internet were included in the regression analyses, which were used to examine the relationship between participant characteristics and having searched for specific categories of infertility health information. Participants were classified into two groups according to treatment duration: those who spent ≤6 months undergoing infertility treatments and those who had been in treatment for >6 months. Treatment duration was dichotomized to determine whether there are differences in the Web-based queries of novice and experienced infertility patients.

### Sample Characteristics

Sample characteristics are presented in Table 1. In the sample of heterosexual couples, nine people did not answer the question about searching the internet; hence, the final sample consisted of 558 participants, including 249 men and 309 women. The mean age was 36.53 (SD 5.5) years and ranged from 22 to 62 years. Approximately half of the sample was born in Canada and had an income below Can $80,000/year. In terms of education, 8.6% (47/548) of participants had completed high school, 25.9% (142/548) obtained a “Collège d’enseignement général et professionnel” diploma (CEGEP, ie, preuniversity and professional training) or technical degree, and 65.5% (359/548) obtained a university degree or more, revealing an overall well-educated group of participants. About one-third of participants reported male factor infertility, whereas another third reported female factor infertility. A small number (44/558, 7.9%) of participants reported diagnoses involving both male and female factors, 17.7% (99/558) of cases were left unexplained, and 5.4% (30/558) of couples had not received diagnostic testing.

**Table 1:** Patients’ demographic characteristics (n=558).

Characteristics		Values, n (%)
**Gender**		
	Male	249 (44.6)
	Female	309 (55.4)
**Annual household income (Canadian dollars)**		
	<$80,000	258 (47.7)
	≥$80,000	283 (52.3)
**Education (highest level achieved)**		
	High school degree or less	47 (8.6)
	Collège d’enseignement général et professionnel/technical college degree	142 (25.9)
	University degree or more	359 (65.5)
**Cause of infertility**		
	Male factor only	180 (32.3)
	Female factor only	194 (34.8)
	Male and female factors	44 (7.9)
	Unexplained	99 (17.7)
	No diagnostic testing	30 (5.4)
**Treatment duration (months)**		
	≤6	218 (39.4)
	>6	335 (60.6)
Born in Canada		288 (52.4)

## Results

### Do Participants Search the Internet?

A majority (490/558, 87.8%) of participants searched the internet for health information about infertility, whereas 12.2% (68/558) of participants did not. On average, those who used Web-based resources endorsed searching for 11 of 20 (SD 3.92) infertility-related topics. Significantly more women (290/309, 93.9%) searched compared with men (200/249, 80.3%; χ^2^_1_=23.6; *P*<.001). Searching the internet was prevalent at all education levels, although those with more education were more likely to search (χ^2^_2_=6.5; *P*=.04); specifically, 89.4% (321/359) of participants with a university degree or more and 88.0% (125/142) of participants with some CEGEP or university degree searched the internet, whereas slightly fewer participants with a high school degree or less (36/47, 76.65%) searched the internet. Participants with longer treatment duration (303/335, 90.4%) were more likely than new patients (184/218, 84.4%) to have searched for Web-based infertility information (χ^2^_1_=4.6; *P*=.03). Perceived stress was associated with searching, with searchers (mean 5.95, SD 2.88) reporting significantly higher levels of perceived stress compared with nonsearchers (mean 5.16, SD 3.02; *t*_555_=–2.11; *P*=.04). Similarly, searchers (mean 1.36, SD 1.40) reported significantly more depressive symptomatology compared with nonsearchers (mean 0.99, SD 1.39; *t*_552_=–2.09; *P*=.04).

### What Do Participants Search for?

#### Descriptive Analysis by Search Category

Out of the 490 participants who searched the internet, all (490/490, 100%) endorsed searching for *diagnostic information*. In addition, nearly all participants endorsed searching for at least one item in the categories *treatment information* (443/490, 90.4%) and *services and providers* (440/490, 89.8%). Approximately two-third of participants (317/490, 64.7%) searched for topics within the category *connections with others*. Table 2 presents a comparison of searching frequencies between men and women for each specific topic of information and support subsumed under the four conceptual categories: *diagnostic information*, *treatment information*, information about *services and providers*, and *connections with others*. Gender and other participant characteristics were examined in relation to search categories.

**Table 2:** Comparison of topics searched by men (n=200) and women (n=290) who reported using the internet to access infertility-related information and support resources.

Types of infertility information		Women, n (%)	Men, n (%)	Chi-square (*df*)	*P* value
**Diagnostic information**					
	Scientific literature about infertility	255 (88.2)	158 (79.0)	7.7 (1)	.006
	Causes of infertility	271 (94.1)	182 (91.0)	1.7 (1)	.19
	Diagnostic tests	225 (78.1)	135 (67.8)	6.5 (1)	.01
	Interpreting results of diagnostic tests	185 (64.5)	199 (59.8)	1.1 (1)	.30
	My diagnosis	223 (77.2)	145 (72.9)	1.2 (1)	.28
**Treatment information**					
	Treatment options	252 (87.5)	169 (84.5)	0.9 (1)	.34
	Medications used in treatment	230 (79.9)	140 (71.1)	5.0 (1)	.03
	Success rates of treatment	242 (83.7)	166 (84.7)	0.1 (1)	.78
	Side effects of treatment	225 (77.9)	143 (72.2)	2.0 (1)	.16
	Using donor sperm or eggs	61 (21.3)	41 (20.9)	0.01 (1)	.91
	Surrogacy	29 (10.1)	27 (13.9)	1.6 (1)	.21
	Adoption/foster parenting	89 (31.1)	52 (26.7)	1.1 (1)	.29
**Information about services and providers**					
	My physician or medical team	200 (69.4)	99 (49.5)	19.8 (1)	<.001
	Clinics where treatment is offered	203 (70.7)	109 (55.6)	11.6 (1)	.001
	How to get a second opinion	69 (24.1)	51 (26.0)	0.2 (1)	.64
	Provincial health care coverage	170 (59.6)	116 (59.2)	0.01 (1)	.92
	Private health care coverage	148 (51.9)	87 (44.6)	2.5 (1)	.12
**Connections with others**					
	How to find peer support/mentor	31 (10.9)	20 (10.2)	0.1 (1)	.81
	Others’ experiences with infertility	199 (69.8)	102 (52.0)	15.7 (1)	<.001
	How to discuss my treatment with family or friends	44 (15.4)	21 (10.7)	2.2 (1)	.14
Other		4 (1.3)	2 (0.8)	31.9 (1)	.67

#### Diagnostic Information

Seeking *diagnostic information* was significantly associated with gender (*t*_479_=–2.32; *P*=.02), such that women (mean 6.43, SD 1.90) searched for more *diagnostic information* compared with men (mean 6.00, SD 2.12). It was further associated with treatment duration (*t*_330.511_=–3.49; *P*=.001), indicating that those who have been in treatment for longer (mean 6.52, SD 1.81) sought more *diagnostic information* than people new to treatment (mean 5.84, SD 2.20). In this category, causes of infertility, scientific literature, and information about diagnosis and diagnostic tests were among the most searched topics for both men and women, although women searched significantly more for scientific literature (255/289, 88.2% vs 158/200, 79.0%) and diagnostic tests (225/288, 78.1% vs 135/199, 67.8%) compared with men (Table 2).

#### Treatment Information

Searching for *treatment information* was significantly related to treatment duration (*t*_475_=–4.19; *P*<.001) only, meaning that people with longer treatment duration (mean 1.90, SD 1.21) searched for more topics related to *treatment information* than people with shorter treatment duration (mean 1.44, SD 1.05). Within the *treatment information* category, treatment options and treatment success rates were among the most searched topics for both men and women, whereas surrogacy and adoption were less commonly searched (Table 2). In addition, more women (230/288, 79.9%) searched for medications used in treatment as compared with men (140/197, 71.1%).

#### Services and Providers

Searching for *services and providers* was associated with gender (*t*_478_=3.52; *P*<.001), with women (mean 2.52, SD 1.25) endorsing searching for more items than men (mean 0.73, SD 0.84). In addition, there was a significant difference between levels of education for searching for that category (*F*_2471_=6.25; *P*=.002). Post hoc Hochberg GT2 tests indicated that those with CEGEP/technical-level (mean 2.40, SD 1.34) and university-level (mean 2.41, SD 1.29) education searched for significantly (*P*=.004 and *P*=.002, respectively) more topics than people with a high school degree or less (mean 1.60, SD 1.24). Searches of people with a CEGEP/technical degree did not differ from those of people with a university degree (*P*>.99). Compared with men, women were significantly more likely to search for information on their physician and medical team (200/288, 69.4% vs 99/200, 49.5%) and for clinics where infertility treatment is offered (203/287, 70.7% vs 109/196, 55.6%; Table 2).

#### Connections With Others

Finally, searching for topics related to *connections with others* was positively correlated with perceived stress (*r*=0.11; *P*=.01) and depressive symptomatology (*r*=0.12; *P*=.01). It was also associated with gender (*t*_396.356_=–3.10; *P*=.002), such that women (mean 0.96, SD 0.78) endorsed more topics in that category compared with men (mean 0.73, SD 0.84). In addition, searching for *connections with others* was related to treatment duration (*t*_474_=–3.44; *P*=.001), suggesting that those with longer treatment duration (mean 0.97, SD 0.82) searched for more *connections with others* than those with shorter treatment duration (mean 0.71, SD 0.77). There was a significant difference between levels of education (*F*_2470_=3.19; *P*=.04). Post hoc Hochberg GT2 tests revealed that those with CEGEP/technical degrees (mean 0.60, SD 0.74) searched significantly more (*P*=.04) for *connections with others* than those with high school diplomas or less (mean 0.98, SD 0.90). However, there was no difference in searching between high school and university (*P*=.20) nor between CEGEP/technical and university (*P*=.38). Others’ experiences with infertility was the most endorsed item in this category, sought by significantly more women (199/285, 69.8%) than men (102/196, 52.0%), whereas the two remaining items about how to find peer support and how to discuss infertility with family and friends were searched less frequently (Table 2).

### Regressions

Regression analysis was used to investigate the unique contribution of the variables that were significantly associated with searching for each of the different categories (Table 3). As only one variable was significantly associated with searching for *treatment information*, no regression analysis was performed for that category. The multiple regression predicting searching for *diagnostic information* was significant (*F*_3467_=7.31; *P*<.001) and explained 4.5% of the variance. More education and longer treatment duration were shown to be significant predictors of seeking *diagnostic information*, whereas gender was not. As for searching for *services and providers*, the significant model (*F*_2471_=8.61; *P*<.001) demonstrated that both being a woman and being more educated were significant predictors of searching for this category, accounting for 3.5% of the variance. As depression and perceived stress were highly correlated with each other (Pearson *r*=0.63; *P*<.001), we tested two separate models predicting the search for *connections with others*. The first model (*F*_3469_=8.12; *P*<.001; *R*^2^=0.049) showed that being a woman, having a longer treatment duration, and feeling depressed were significant predictors of looking on the internet for resources regarding connections with others. The second model (*F*_3471_=8.06; *P*<.001; *R*^2^=0.049) also indicated that gender and treatment duration were significant determinants but perceived stress was not.

**Table 3:** Regression analysis summary for participant characteristics associated with searching for diagnostic information, services and providers, and connections with others.

Variables		Unstandardized beta	SE for unstandardized beta	Standardized beta	*t* value (*df*)	*P* value
**Diagnostic information**						
	Gender	.35	.18	.09	1.88 (467)	.06
	Education	.34	.15	.11	2.36 (467)	.02
	Treatment duration	.60	.19	.15	3.23 (467)	.001
**Services and providers**						
	Gender	.39	.12	.15	3.26 (471)	.001
	Education	.23	.10	.11	2.43 (471)	.02
**Connections with others - model 1**						
	Gender	.20	.08	.12	2.61 (469)	.01
	Treatment duration	.24	.08	.14	3.12 (469)	.002
	Depression	.05	.03	.09	2.00 (469)	.05
**Connections with others - model 2**						
	Gender	.20	.08	.12	2.63 (471)	.01
	Treatment duration	.24	.08	.14	3.12 (471)	.002
	Perceived stress	.02	.01	.08	1.77 (471)	.08

### Meeting the Needs of Patients

Of those who searched on the internet, 70.9% (346/488) indicated that their needs were met and 29.1% (142/488) indicated that their needs were not met by Web-based information. Table 4 represents the proportions of participants who searched for specific topics of information depending on whether their needs were or were not met. Participants who reported having their needs met by Web-based resources were more likely to search for information about the following topics: their diagnosis, treatment options, success rates of treatment, provincial health care coverage, how to find peer support/mentor, and how to discuss treatment with family or friends. With respect to the broader categories, those with met needs (mean 6.42, SD 1.94) reported searching for more items in the *diagnostic information* category compared with those with unmet needs (mean 5.85, SD 2.11; *t*_478_=–2.87; *P*=.004); there was no difference between groups for the three other categories.

**Table 4:** Comparison of topics searched by participants reporting met (n=346) and unmet (n=142) needs.

Types of infertility information			Met needs, n (%)		Unmet needs, n (%)		Chi-square (*df*)		*P* value
**Diagnostic information**									
	Scientific literature about infertility	296 (85.5)		117 (82.4)		0.8 (1)		.38	
	Causes of infertility	323 (93.4)		129 (91.5)		0.5 (1)		.47	
	Diagnostic tests	263 (76.2)		96 (68.1)		3.4 (1)		.06	
	Interpreting results of diagnostic tests	223 (64.8)		80 (56.7)		2.8 (1)		.10	
	My diagnosis	271 (78.6)		96 (67.6)		6.5 (1)		.01	
**Treatment information**									
	Treatment options	307 (88.7)		113 (80.1)		6.2 (1)		.01	
	Medications used in treatment	266 (77.3)		103 (73.6)		0.8 (1)		.38	
	Success rates of treatment	300 (87.7)		108 (76.1)		10.3 (1)		.001	
	Side effects of treatment	268 (77.9)		99 (69.7)		3.6 (1)		.06	
	Using donor sperm or eggs	75 (21.9)		27 (19.4)		0.4 (1)		.54	
	Surrogacy	37 (10.9)		19 (13.7)		0.7 (1)		.39	
	Adoption/foster parenting	94 (27.6)		47 (33.8)		1.9 (1)		.17	
**Information about services and providers**									
	My physician or medical team	213 (61.6)		85 (60.3)		0.1 (1)		.79	
	Clinics where treatment is offered	222 (64.9)		90 (64.3)		0.02 (1)		.9	
	How to get a second opinion	89 (26.0)		31 (22.3)		0.7 (1)		.39	
	Provincial health care coverage	215 (62.9)		71 (51.1)		5.7 (1)		.02	
	Private health care coverage	172 (50.4)		63 (45.3)		1.0 (1)		.31	
**Connections with others**									
	How to find peer support/mentor	43 (12.6)		8 (5.8)		4.8 (1)		.03	
	Others’ experiences with infertility	212 (62.2)		89 (64.0)		0.1 (1)		.7	
	How to discuss my treatment with family or friends	53 (15.5)		12 (8.6)		4.0 (1)		.046	
Other			3 (0.9)		3 (2.1)		48.0 (1)		.09

Only the psychological distress variables were found to be significantly related to having unmet needs for information. Participants whose needs were unmet (mean 6.51, SD 2.89) reported greater perceived stress than participants whose needs were met (mean 5.72, SD 2.85; *t*_485_=2.80; *P*=.005). Similarly, people with unmet needs (mean 1.58, SD 1.53) reported significantly more depressive symptomatology compared with those with met needs (mean 1.28, SD 1.35; *t*_482_=2.18; *P*=.03). Gender, education level, and treatment duration were unrelated to having met or unmet needs.

## Discussion

### Principal Findings

To determine whether there is a need to tailor Web-based health resources to support infertility patients, this study sought to identify the kind of information users were seeking and whether existing resources met their needs. Survey results revealed a nearly ubiquitous search for Web-based information about infertility diagnosis, treatment, services, and providers, and many respondents also sought online emotional support and access to others. Web-based resources were generally perceived to meet the searchers’ needs, and there was little evidence of a digital divide, in that there were no differences in search patterns by income or immigrant status. However, certain factors were associated with distinct search patterns and satisfaction with Web-based resources; these included gender, infertility treatment duration, and psychological distress.

Although men and women searched for similar types of information, women were more likely to search the internet for scientific literature, their physician or medical team, diagnostic tests, medications used in treatment, clinics where treatment is offered, and for others’ experiences with infertility, demonstrating women’s greater interest in gathering both practical and experiential information about infertility. This finding is in line with research showing that women engage in more Web-based health information seeking than men [35] and that they are more active in online support groups [37]. Women also tend to assume primary responsibility for gathering information about infertility [10]. As most treatment procedures are centered on women’s bodies regardless of the cause of infertility, it makes sense that women take a greater interest in searching for information such as medications used in treatment. Men, on the other hand, often feel excluded from the infertility treatment experience [52]. Furthermore, a recent review of Canadian infertility websites revealed that only 20% of fertility clinic websites included information about male infertility [25], indicating that Web-based resources may be more targeted to women. This study did not identify any specific topic searched more by men compared with women. Given that the current findings indicate that some men are in fact searching for infertility information on the internet, and other research suggests that men also benefit from experiential information found on online forums [39], further investigation of the specific interests of male infertility patients is warranted.

Treatment duration was associated to more searching for information about diagnosis and treatment, suggesting that participants who had spent more time in treatment may have been looking for alternatives because of treatment failures. In addition, those with longer treatment duration sought more connections with others, which may indicate a greater need for emotional support following a prolonged treatment process. Seeking online social support may benefit infertility patients by providing a medium for normalizing and validating infertility-specific problems and reducing social isolation during this difficult time.

This study addressed an important gap in the literature regarding the ability of Web-based resources to meet infertility patients’ needs [12], given the variability of Web-based infertility-related resources in terms of quality, readability, and suitability [24,25]. In this study, the majority of participants found Web-based resources useful in meeting their informational and support needs. As infertility patient populations tend to be fairly well educated, they may have the eHealth literacy and searching skills necessary to find and access the infertility resources that correspond to their needs. Indeed, the results of this study indicated that less well-educated patients were less likely to search the internet, though there was no difference by education in reporting that the resources met their needs. Alternative sources of information such as written materials (ie, pamphlets) and in-person discussions may be preferred by patients with lower levels of education. Further investigation of the relationship between level of education and searching the internet is needed to understand the nature of this correlation.

Although people with higher levels of psychological distress were more likely to search for Web-based sources of information, they more often reported unmet needs for information and support. Psychological distress was the only factor that distinguished the almost one-third of participants who reported that Web-based information and support resources did not meet their needs. It is well established that infertility can be a source of psychological distress for both men and women [36]. It may be that greater perceived stress and depression triggers patients to rely more on the internet to access informational and emotional support resources. In support of this, previous research has identified a number of psychological factors associated with increased online health seeking behaviors including health anxiety [46], neuroticism [53], dissatisfaction with care [54], and perceptions of poor health [3]. Our results also show that distressed participants sought more social support resources than those without distress. This suggests that people experiencing infertility-related stress and depression have an increased desire to connect with people who understand their situation compared with those who are less distressed and that Web-based resources may facilitate those connections.

Despite the fact that they searched more for Web-based resources about infertility, our findings indicate that the likelihood of unmet needs increased with greater stress and depressive symptoms. It is possible that Web-based resources are not tailored to the needs of those with high levels of infertility-related distress. Future research should investigate the types of resources that people with high levels of infertility-related distress want and work with them to develop those resources. On the other hand, it may also be that the internet caused distress in certain cases or that distressed people viewed things more negatively [55]. For example, Malik and Coulson [20] found that participating in online support groups can expose users to stories about negative experiences with infertility, pregnancy news, and inaccurate information, which may be upsetting to infertility patients. In other cases, people may not succeed in finding or accessing the information and support resources that they are looking for on the internet, thereby feeling more distressed than before the search. Research shows that patients who have difficulty understanding or filtering available Web-based information are likely to experience information overload, which has been associated with poorer psychological well-being [56]. In contrast, participants with better mental health may have been more likely to report met needs because they were not looking for information regarding mental health and how to cope with infertility. However, this study did not explicitly ask participants why internet resources did or did not meet their needs nor whether the resources included an information category regarding mental health and coping. Future studies should inquire directly about the reasons why certain infertility patients are dissatisfied with the information and support resources available on the internet and why other patients feel that the internet appropriately meets their needs. This is an important step for tailoring Web-based resources to better meet the needs of those with infertility concerns. It is also possible that other sources of information and support, such as health care professionals, counselors, and in-person peer support groups, may be better suited to meet the needs of people in distress. A recent study found that the perceived stress of women participating in online support groups for infertility was unrelated to the supportive elements gained from those groups [57], suggesting that other forms of support may be required to relieve their stress.

### Strengths and Limitations

One of the strengths of this study is the recruitment of participants from fertility clinics rather than through online infertility forums. This allowed for greater generalizability of the results as it does not restrict the sample to only those who use online communities, thereby assessing how a diverse sample of infertility patients use different Web-based health resources and allowing the investigation of the characteristics of those who do not search the internet. In addition, this study inquired about a comprehensive list of topics that infertility patients are likely to look for. Furthermore, this study extended the literature by having a sample that included not only women but also an almost equal number of men. Including men in fertility research is important, as men also go through the challenges involved in infertility diagnosis and treatment as well as experience infertility-related distress [58]. Although more women than men reported that they had searched for Web-based infertility resources, most men still used the internet to seek infertility information and support.

Notwithstanding several study strengths, limitations must be noted. First, the participants of this study included only people seeking infertility care; therefore, this sample may not be representative of people with infertility who are not seeking medical treatment. Furthermore, the recruitment procedure involved a self-selection process, which may introduce bias among study participants as those individuals who volunteered to take our Web-based survey may be more comfortable using the internet. Although a large majority (81.15%) of people in the United States have access to the internet according to a recent survey, internet access remains associated with sociodemographic factors such as age lower than 35 years, non-Hispanic white ethnicity, higher education, higher income, and urban residency [59]. It should be noted that in this study, all those who were approached to solicit their participation met the inclusion criterion of having internet access. In addition, these analyses included heterosexual couples only. Future studies should explore the online experiences and satisfaction of a more diverse sample of people with infertility concerns by including single and nonheterosexual individuals. This would allow infertility researchers and care providers to better understand the distinct needs and concerns of single individuals and same-sex couples who are increasingly using assisted reproduction techniques to fulfill their family plans. For example, it would be important to understand the extent to which Web-based resources meet the needs for social connection in these groups as well as information regarding such topics as donor gametes and surrogacy.

Although it was found that participants with met needs searched significantly more for medical information about diagnosis and treatments as well as for proactive social support resources, this study did not identify any type of Web-based infertility information and support that was searched more by those with unmet needs. Although our list of items was comprehensive, it is possible that these participants searched for types of resources that were not included in the response options. It is also possible that other participant characteristics, such as low health literacy [60], may have made it less likely that participants would find the right information for their needs or be satisfied with the information found.

Finally, data analysis was primarily performed using correlational statistics, which, although suitable to identify statistical relationships between variables, does not allow detection of the directionality of relationships or to make causal interpretations. Thus, we cannot draw conclusions about whether distress caused participants to have unmet needs or whether having unmet needs caused distress.

### Implications for Future Research

Longitudinal research is needed to examine the direction of the relationship between experiencing psychological distress, searching the internet, and having needs met or unmet by the internet. In addition, qualitative interviews or questionnaires could be used to determine why infertility patients believe Web-based information did or did not meet their needs. This information may help to inform the development of Web-based resources with appropriate information and support tools that correspond to the needs of infertility patients and of those not in treatment but who require information and support in the process of trying to conceive. The vastness, inconsistency, and variable quality of Web-based resources may act as a barrier for obtaining infertility information and support. Future research should explore whether individualization and tailoring of Web-based resources can improve the perceived met needs and well-being of infertility patients, especially those who experience distress. Emergent evidence suggests that the development of patient-centered psychoeducational eHealth interventions may provide an opportunity to empower people with infertility problems and to address their needs for information and support [61-65]. However, the eHealth literature could benefit from more research to assess the feasibility and usability of these interventions as well as the informational and emotional advantages and the cost-effectiveness of these potential solutions [66].

### Conclusions

Both men and women undergoing infertility treatment used the internet to seek a broad range of information and support resources related to infertility. Most of them reported that Web-based resources met their needs. Distressed patients reported particularly high rates of searching, but their needs were not always met. Further research is needed to understand why distressed patients do not find Web-based resources helpful in meeting their needs and whether alternative ways of providing information and support may be better suited for these people. This study reveals that most infertility patients are well able to find Web-based resources that meet their needs. However, certain infertility patients may benefit from discussions with their health care providers regarding the use of the internet for obtaining information and support; providers may be able to help them navigate through the large number of infertility-related resources by suggesting trustworthy and suitable websites.

## Appendix

Multimedia Appendix 1 The patient survey.

## Abbreviations

CEGEP: Collège d’enseignement général et professionnel
eHealth: electronic health
