# Supplementary material for: Searching the Internet for Infertility Information: A Survey of Patient Needs and Preferences
Source: J Med Internet Res. 2019 Dec 12;21(12):e15132. doi: 10.2196/15132 (PMC6933516; doi:10.2196/15132)
Supplement: Multimedia Appendix 1 [file jmir_v21i12e15132_app1.pdf]

Thank you for agreeing to take part in our study. Our goal is to better understand the information and support needs of fertility patients. The survey should take approximately 15-20 minutes to complete. We greatly appreciate your input and respect your privacy. All answers are separated from your e-mail address, to ensure anonymity and confidentiality. When we analyze the data, the responses of all participants will be grouped together.

We would like to know about your fertility history

---

1. **Please indicate your sex.**
   a. Male
   b. Female

2. **How many children do you have?**
   a. 0 (Q3 does not appear)
   b. 1
   c. 2
   d. More than 2

3. **How many of these children were:**
   A. Conceived naturally (Drop-down: 0, 1, 2 more than 2)
   B. Conceived with the help of infertility treatment/assisted reproductive technologies (0, 1, 2 more than 2)
   C. Adopted (0, 1, 2 more than 2)
   D. Stepchildren (0, 1, 2 more than 2)
   E. Other _______________ (0, 1, 2 more than 2)

4. **How long were you trying to conceive prior to seeking medical treatment?**
   a. Less than 6 months
   b. 6 months to 1 year
   c. 1 to 2 years
   d. 3 to 5 years
   e. More than 5 years

5. **How long have you been receiving fertility treatment?**
   a. Less than 6 months
   b. 6 months to 1 year
   c. 1 to 2 years
   d. 3 to 5 years
   e. More than 5 years

6. **What is the cause of your infertility?*(check all that apply)***
   a. Abnormal Sperm Morphology
   b. Anovulation
   c. Advanced maternal age
   d. Blocked or impaired fallopian tubes
   e. Diminished ovarian reserve
   f. Endometriosis

g. Uterine Malformations
h. Uterine Fibroids
i. Polycystic Ovarian Syndrome
j. Infertility due to cancer treatment or other medical conditions
k. Low semen count
l. Penile or other genital malformations
m. Reduced Sperm Motility
n. Varicocele
o. Vasectomy
p. Unexplained
q. Other ____

7. What kinds of fertility treatment(s) have you received? (*check all that apply)*
(note all terms will be defined in a hover over box)
a. This is my first visit
b. Initial consult
c. Treatment with oral hormones for women (e.g. Clomiphene, Metformin)
d. Treatment with oral hormones for men (e.g. hCG, hMG)
e. Treatment with injectable hormones (e.g. FSH/Gonadotropin)
f. Vaginal, cervical or intrauterine insemination (IUI)
g. Intra-cytoplasmic Sperm Injection (ICSI)
h. Tubal surgery
i. In vitro fertilization (IVF)
j. Embryo transfer
k. Gamete Intra-fallopian Transfer (GIFT)
l. Egg donation
m. Surrogacy
n. Laparoscopic Surgery
o. Percutaneous Sperm Aspiration (PESA)
p. Testicular Sperm Aspiration (TESA)
q. Testicular Sperm Extraction (TESE)/Surgical Sperm Retrieval (SSR)
r. Varicocele treatment
s. Vasectomy reversal (e.g. vasoepididymostomy, vasovasostomy)
t. Cryo-preservation (Sperm banking)
u. Other ___________________________

8. These questions ask you about your feelings and thoughts **during the last month**. In each case, please indicate how often you felt or thought a certain way.
(scale: 0 = never, 1 = almost never, 2 = sometimes, 3 = fairly often, 4 = very often)

- In the last month, how often have you felt that you were unable to control the important things in your life?
- In the last month, how often have you felt confident about your ability to handle your personal problems?
- In the last month, how often have you felt that things were going your way?
- In the last month, how often have you felt difficulties were piling up so high that you could not overcome them?

9. Over the past two weeks, how often have you been bothered by any of the following problems? (scale: 0 = not at all, 1 = several days, 2 = more than half the days, 3 = nearly every day)

- Little interest or pleasure in doing things
- Feeling down, depressed or hopeless

10. Have you sought psychological counselling during the course of fertility treatment? (yes, no) *(branching)*
If no: Would you have liked to have access to psychological counselling? (yes, no)

We would like to know how you use the internet to find health information.

---

11. What internet access do you have? (check all that apply)
a. No internet access
b. Wired and/or wireless (Wi-Fi) internet access at home
c. Data plan for internet access on a mobile phone or tablet
d. Wireless (Wi-Fi) access on a mobile phone or tablet
e. Internet access at work or school or public locations

12. Have you searched online for information about fertility?

a. Yes (branching occurs to below)
b. No (goes to 19)

13.

**Did you look online for information about:**	***Yes***	***No***
Scientific literature on infertility		
My attending physician or medical team		
Causes of infertility		
Diagnostic tests		
Interpreting results of diagnostic tests		
My diagnosis		
Treatment options		
Medications used in treatment		

Success rates of treatment		
Side effects of treatments		
Using donor sperm or eggs		
Surrogacy		
Adoption/Foster parenting		
Clinics where treatment is offered		
How to get a second opinion		
How to find peer support/mentor		
Others' experiences with infertility		
What was covered by my provincial health care plan		
What was covered by my private health care plan		
How to discuss my treatment with family or friends		
Other ___________________________________		

14. In general, did the information you found online meet your needs?

a. Yes
b. No

15. Which website(s) do you prefer to consult about fertility?

____

We would like to know a little about you

---

23. What is your age?

(drop down list 18-75)

24. Which group best represents your ethnic origin?

a. Aboriginal (e.g., First Nations, Inuit, Métis)
b. Arab
c. Black
d. Chinese
e. Filipino

f. Japanese
g. Korean
h. Latin American
i. South Asian (e.g. Indian, Pakistani, Bangladeshi, Sri Lankan)
j. Southeast Asian (e.g. Cambodian, Indonesian, Laotian, Vietnamese)
k. West Asian (e.g. Afghan, Iranian)
l. White
m. Other group (please specify) _________________

25. What is the language you speak most often at home?
a. English
b. French
c. Other (please specify) _________________

26. Were you born in Canada?
a. Yes
b. No (if no, Q29 and Q30 appear)

27. Where were you born?
(list of regions drop-down)
a. Africa
b. Asia
c. Central America
d. Eastern Europe
e. Western Europe
f. Middle East
g. North America
h. Oceania (Australia, Philippines and surrounding islands)
i. South America
j. The Caribbean

28. How many years have you lived in Canada?
(drop down menu)
__________years

29. Are you a:
a. 1st Generation Immigrant (You were born outside of Canada)
b. 2nd Generation Canadian (At least one of your parents was born outside of Canada)
c. 3rd Generation Canadian and Beyond (Both you and your parents were born in Canada)
d. Other_______________

30. Do you consider yourself as belonging to any particular religion?
a. Yes (branching – Q11 appears)
a. No

31. If yes, which one? (drop down menu alphabetical)
a. Anglican/Church of England
b. Atheist/agnostic/None

c. Bahá'í Faith
d. Baptist
e. Brethren
f. Buddhism
g. Caodaism
h. Christian—no denomination
i. Daoism
j. Free Presbyterian
k. Hinduism
l. Islam/Muslim
m. Jainism
n. Jehovah's Witness
o. Jewish
p. Korean Shamanism
q. Lutheran
r. Mennonite
s. Methodist
t. Mormon
u. Presbyterian/Church of Scotland
v. Roman Catholic
w. Seventh-Day Adventist
x. Shinto
y. Sikhism
z. Taoism
aa. United Church of Canada
bb. United Reform Church/Congregational
cc. Other (please specify) ______________________

32. Please indicate your employment status:

a. Part-time
b. Full-time
c. Full-time student
d. Unemployed due to illness or disability
e. Unemployed due to other reasons
f. Parental leave and/or stay at home parent
g. Retired
h. Other__________________________

33. Which income bracket best describes your annual household income?

a. Less than $20,000
b. $20,000 to $39,000
c. $40,000 to $59,000
d. $60,000 to $79,000
e. $80,000 to $99,000
f. $100,000 to $120,000
g. More than $120,000

34. What is the highest level of education you have completed?

a. Less than high school
b. Completed some high school
c. High school graduate or equivalent
d. Technical college, community college or CEGEP
e. Completed some university, but no degree
f. University graduate
g. Completed some post-graduate but no degree
h. Completed post-graduate school (e.g., MSc., M.D., PhD)

35. In describing your sexual orientation, do you consider yourself to be

a. Heterosexual or straight
b. Gay or lesbian
c. Bisexual
d. Other (please specify) _________________________

36. What is your current marital status?

a. Single
b. Long-term dating
c. Living with a common law spouse
d. Married
e. Separated
f. Divorced
g. Widower/widow

37. Did you find any of these questions stressful, inappropriate or invasive?

a. Yes (if yes, which ones?)
b. No

38. If you have any questions, comments or suggestions regarding the information presented here, feel free to contact [contact information here]
